# The Response of *Picea abies* Somatic Embryos to UV-B Radiation Depends on the Phase of Maturation

**DOI:** 10.3389/fpls.2018.01736

**Published:** 2018-11-27

**Authors:** Kateřina Eliášová, Zuzana Vondráková, Lenka Gemperlová, Vilém Neděla, Jiří Runštuk, Lucie Fischerová, Jiří Malbeck, Alena Trávníčková, Milena Cvikrová, Martin Vágner

**Affiliations:** ^1^Institute of Experimental Botany of the Czech Academy of Sciences, Prague, Czechia; ^2^Institute of Scientific Instruments of the Czech Academy of Sciences, Brno, Czechia

**Keywords:** ferulic acid, *Picea abies* (L.) Karst., polyamines, somatic embryogenesis, viability

## Abstract

Ultraviolet-B (UV-B) radiation is a key environmental signal which initiates diverse responses that affect the metabolism, development, and viability of plants. In keeping with our previous studies, we concentrated primarily on how UV-B radiation affects Norway spruce [*Picea abies* (L.) Karst.] somatic embryo maturation and how phenolics and polyamines (PAs) are linked to the defense response invoked by UV-B irradiation. We treated clusters of Norway spruce embryogenic culture (EC) with UV-B during the five stages of embryo maturation (early, cylindrical, precotyledonary, cotyledonary, and mature embryos). For the first time, we take an advantage of the unique environmental scanning electron microscope AQUASEM II to characterize somatic embryos in their native state. The severity of the irradiation effect on embryonal cell viability was shown to be dependent on the intensity of radiation as well as the stage of embryo development, and might be related to the formation of protoderm. The response of early embryos was characterized by an increase in malondialdehyde (MDA), a marked decrease in PA contents and a decline in phenolics. The reduced ability to activate the defense system seems to be responsible not only for the severe cell damage and decrease in viability but also for the inhibition of embryo development. The significant reduction in spermidine (Spd), which has been reported to be crucial for the somatic embryo development of several coniferous species, may be causally linked to the limited development of embryos. The pronounced decrease in cell wall-bound ferulic acid might correspond to failure of somatic embryos to reach more advanced stages of development. Embryos at later stages of development showed stress defense responses that were more efficient against UV-B exposure.

## Introduction

Ultraviolet-B (UV-B) radiation affects plants both directly and indirectly, and can, for example, damage DNA, proteins, and membranes, alter transpiration and photosynthesis, and lead to changes in growth, development, and morphology (Jansen et al., 1998). Oxidative stress is a common response to unfavorable environmental conditions such as UV radiation (Hideg et al., 2013). The scavenging of active oxygen and other radical species, either through enzymatic or non-enzymatic systems, can alleviate UV stress (Jansen et al., 1996).

Polyamines (PAs), which constitute a group of low molecular weight aliphatic amines, are key to regulating growth and developmental processes in plants, as well as the response to biotic and abiotic stresses (Kusano et al., 2008; Gill and Tuteja, 2010; Takahashi and Kakehi, 2010; Hussain et al., 2011). Studies using loss-of-function mutants and transgenic plants to modulate PA metabolic pathways have confirmed that PAs play a protective role during plant responses to abiotic stress (Shi and Chan, 2014). Possibly the radical-scavenging activity of PAs moderates UV-B radiation stress, as has been demonstrated for other free-radical scavengers (Jansen et al., 1996). Moreover, species-specific and age-dependent differences in the quantitative and qualitative composition of PAs could affect the susceptibility of a plant to abiotic stresses (Reifenrath and Müller, 2007).

One of the most frequently observed responses to increased UV-B exposure is the activation of the phenylpropanoid biosynthetic pathway (Takshak and Agrawal, 2015). Many phenylpropanoids have both antioxidant and UV-B screening properties (Jansen et al., 2008; Agati and Tattini, 2010), and mutants deficient in the accumulation of these compounds (e.g., *Arabidopsis transparenta testa*) are considerably more susceptible to UV-B radiation than the corresponding wild-type lines (Booij-James et al., 2000). Phenolic compounds are generally viewed as harmful for *in vitro* cultures (both in micropropagation and somatic embryogenesis) since their exudation and oxidation negatively affects explants, causing browning and necrosis, especially when mature explants of woody plants are used (Martin and Madassery, 2005). However, Reis et al. (2008) provided evidence that phenolic compounds are involved in certain *in vitro* morphogenic processes, including the induction of somatic embryogenesis in *Feijoa sellowiana*. Somatic embryos were formed in the proximity of phenolic-rich cells which, in more advanced stages of development, formed a zone isolating the embryo from the maternal tissue. Furthermore, according to Cvikrová et al. (2008, 2010), phenolic compounds participate in resistance mechanisms that are important for *P. abies* ECs.

We have previously reported that the accumulation of PAs in fully developed *P. abies* somatic embryos may be causally linked to better tolerance of UV-B irradiation (Cvikrová et al., 2016). The research presented in this paper focused on the variable sensitivity of an EC to UV-B radiation during the course of maturation. More specifically, the research aimed to determine whether irradiation affects the development of spruce somatic embryos and to evaluate the ability of an EC to activate the stress defense response upon UV-B exposure. We exposed EC clusters to UV-B during the embryo maturation. The effects of UV-B irradiation were described on the morphological and biochemical levels. We used the unique environmental scanning electron microscope AQUASEM II, which enables the observation of beam sensitive samples as wet biopolymers (Schenkmayerová et al., 2014), plant extracellular matrix (Neděla et al., 2012), or plant waxes (Neděla et al., 2015b) in their native states, to characterize the developmental stages. This permitted us to study how the pattern of surface cell layers, which is crucial to plant defenses against radiation, develops. The morphological evaluation methods included light, fluorescence, and electron microscopy, while the biochemical analyses focused on monitoring changes in the contents of PAs and phenolic acids.

## Materials and Methods

### Plant Material

An EC of Norway spruce (*Picea abies* L. Karst.), genotype AFO 541, was obtained from AFOCEL (Nangis, France). The cultivation of the EC followed a protocol described by Cvikrová et al. (2016). During proliferation, the EC was cultivated in plastic Petri dishes (10 cm in diameter) on GD medium (Gupta and Durzan, 1986) solidified with 0.75% agar and supplemented with sucrose (30 g/L) and the phytohormones 2,4-D (5 μM), kinetin (2 μM), and BAP (2 μM) (all Duchefa, Haarlem, Netherlands). To initiate the maturation phase, cytokinins and auxin in the medium were replaced with abscisic acid (20 μM) and 3.75% polyethylene glycol 4000 (all Sigma-Aldrich, St. Louis, MO, United States). The cultures were kept in darkness at 23 ± 1°C during both proliferation and maturation and were subcultured onto fresh medium once per week.

#### UV-B Irradiation and Collection of Plant Material for Analysis

Closed plastic Petri dishes with cultures were exposed to UV-B irradiation at the third day after regular 1-week subcultivation in the first, second, third, fourth, and fifth weeks of maturation. These time points corresponded to stages I, II, III, IV, and V of embryo development. UV-B treatment was performed in a box (BIO-LINK, BLX-312, Vilber Lourmat, France) equipped with 312 nm UV lamps. Each treatment represented one short-term exposure (15 and/or 75 s of UV-B radiation). Precise measurement of the amount of UV energy that samples were exposed to was ensured by a UV sensor cell within the radiation chamber. The exposures, 15 and 75 s, are equivalent to intensities of 0.1 and 0.6 W m^-2^, respectively, acting for 1 h. The transmittance of the plastic Petri dishes was measured on a defined wavelength (312 ± 2 nm) and statistically evaluated [*T* = (63 ± 0.6)%]. The average transmittance was then used to recalculate the UV-B dose that the ECs had been exposed to.

In order to avoid the effect of UV-B irradiation on media composition, the ECs were transferred onto fresh medium and returned to the original light regime immediately after UV-B treatment. The same cultivation regime was applied to the control variant.

Material from two independent experiments was analyzed at anatomical and biochemical level. At least four biological replicates of control (non-treated) and irradiated ECs were collected for malondialdehyde (MDA), PA, and phenolic acid analyses on the seventh day after treatment. The control and irradiated ECs were further analyzed for phenolic acids at the end of maturation (i.e., after 6 weeks). Material from each variant was dried on cotton wool, divided into samples and frozen in liquid nitrogen. The samples were stored at -80°C until the biochemical analysis.

### Microscopic Analysis

#### Specification of Developmental Stages

The developmental steps of spruce somatic embryos were documented on the morphological and anatomical levels using a Jenaval transmission light microscope (Zeiss, Jena, Germany) after staining with 0.4% Trypan Blue (Sigma-Aldrich) as described elsewhere (Vondráková et al., 2014).

#### The Yield of Embryos

Small clusters (0.1 g FW) of EC were placed on medium in Petri dishes at the start of maturation and whole intact clusters were weekly subcultured. The number of mature embryos was estimated after 6 weeks of maturation from 10 clusters corresponding to 1 g of EC inoculated. Images of 10 clusters per variant that contained mature embryos were recorded and the total number of embryos (including those that were malformed or only partially developed) in each image was counted, along with the number of fully developed mature embryos.

An SMZ 1500 stereomicroscope (Nikon, Tokyo, Japan) was used to observe EC morphology and determine embryo yield. All of the images were recorded using a Nikon DS-5M digital camera and processed using the Nis-Elements AR 3.2 (Laboratory Imaging, Prague, Czechia) computer image analysis system.

#### Environmental Scanning Electron Microscopy (ESEM)

A non-commercial environmental scanning electron microscope AQUASEM II (Institute of Scientific Instruments of the CAS, Brno, Czechia) equipped with self-developed ionization detectors of secondary electrons (Neděla et al., 2015a) was used for the micro-morphological description of the embryo surface and to study how embryos – in their native state without any chemical fixation, dehydration, or coating – change during maturation. Norway spruce somatic embryos were placed onto the water-cooled Peltier stage with a silicone surface. Before observation with the ESEM, samples with dimensions of 3–6 mm^2^ and a thickness of 2 mm were rinsed with distilled water for 20 min to minimize and dissolve the mucous layer. Before specimen chamber evacuation, samples were put into a drop of 2 μl of distilled water to both obtain better thermal contact between the sample and the Peltier stage and to achieve better sample hydration. The working conditions for observations were: water vapor pressure from 500 to 580 Pa; sample temperature -1°C; beam energy 20 keV; beam current 60 pA; and environmental distance 3–4 mm.

#### Viability Assay

Viability assay was performed at the first, second, third, fourth, and fifth weeks of maturation on the seventh day after irradiation with 0.1 and 0.6 W m^-2^ h^-1^ UV-B and in the corresponding un-treated samples. Cell viability was determined using double staining with fluorescein diacetate (FDA) and propidium iodide (PI) (both Sigma-Aldrich) according to a modified version of the protocol presented by Vondráková et al. (2010). A small piece of EC was suspended in cultivation medium and stained with 2.25 μM PI (stock solution 1 mg mL^-1^ H_2_O) and freshly diluted 4.8 μM FDA (stock solution 2 mg mL^-1^ acetone). Within 5 min, the samples were observed using an LSM 5 Duo confocal laser-scanning microscope (Zeiss) equipped with an Argon/2 laser (FDA excitation at 488 nm, emission filter-set BP 505–550) and a DPSS laser (PI excitation at 561 nm, emission filter-set LP 650). The viable cells were observed based on the enzymatic hydrolysis of FDA to form fluorescein, which exhibits bright green fluorescence. PI intercalates with nucleic acids of non-viable or dead cells to form complexes that emit bright red fluorescence. All images are shown at maximum intensity projections of confocal optical sections of the whole embryos.

### Biochemical Analyses

#### Polyamine Analysis

The extraction and HPLC/MS analysis of benzoylated PAs was performed as described by Eliášová et al. (2017).

#### Phenolic Acid Analysis

Phenolic acids were extracted as described by Cvikrová et al. (1991). Briefly, free, ester-bound (those released after alkaline hydrolysis) and glycoside-bound (those released after acid hydrolysis) phenolic acids were obtained from a methanol extract of tissue ground in liquid nitrogen. The cell wall-bound phenolic acids were obtained through alkaline hydrolysis of the residual material following methanol extraction. The detection and quantification of phenolic acids was carried out using an HPLC/MS system as described by Eliášová et al. (2017).

#### Malondialdehyde Assay

The MDA content of the samples was determined using an NWLSS-Malondialdehyde Assay kit (cat. no. NWK-MDA01, Northwest Life Science Specialties LLC, Vancouver, BC, Canada) as described in detail by Cvikrová et al. (2013).

### Statistical Analysis

The mean ± SE of two independent experiments with a minimum of four biological replicates per experiment are shown in the figures. Standard descriptive statistics were used when analyzing the data. To assess the significance of between-treatment differences, we used a two-step procedure: a preliminary *F*-test of standard deviations (classical *F*-test hypothesis H_0_: σ_1_^2^ = σ_2_^2^, *H*_1_: σ_1_^2^ ≠ σ_2_^2^; *R* < *F* α/2) and t-test of averages (hypothesis H_0_: μ_1_^2^ = μ_2_^2^, H_1_: μ_1_^2^ ≠ μ_2_^2^; |*R*| < t α). Asterisks above bars indicate significant differences (*P* < 0.05) between the contents observed in irradiated somatic embryos and the contents observed in the corresponding controls.

## Results

### Description of Somatic Embryo Development

**FIGURE 1:**
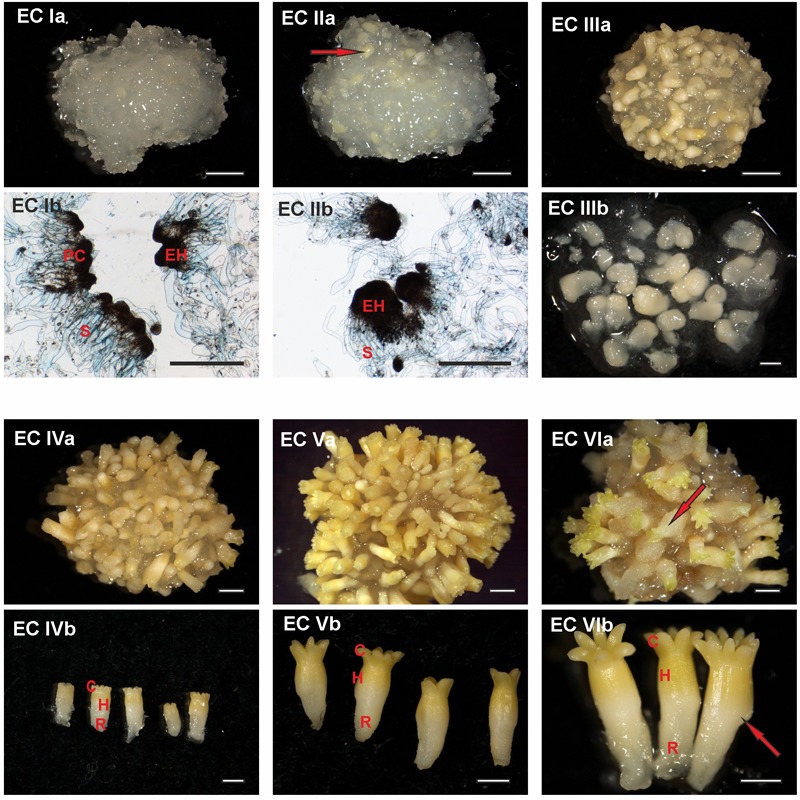
Documentation of *Picea abies* somatic embryos at distinct phases of somatic embryo maturation. **(EC Ia)** Cluster of embryogenic suspensor mass during the first week of maturation; **(EC Ib)** detailed morphology of early embryos, with individual embryonal heads (EH) connected to elongated suspensor cells (S); large polyembryogenic centers, i.e., meristematic zone from which embryonal heads can be separated (PC). **(EC IIa)** Cluster of embryogenic suspensor mass during the second week of maturation (the arrow highlights a somatic embryo that is visible on the surface of the cluster); **(EC IIb)** detailed morphology of elongated early embryos, with enlarged embryonal heads (EH) still connected to the suspensor (S). **(EC IIIa)** Cluster of embryogenic suspensor mass with visible somatic embryos during the third week of maturation; **(EC IIIb)** detailed morphology of precotyledonary embryos. **(EC IVa)** Cluster of maturing somatic embryos during the fourth week of maturation; **(EC IVb)** detailed morphology of cotyledonary embryos demonstrating a circle of cotyledons (C), hypocotyl (H) and radicle (R). **(EC Va)** Cluster of mature somatic embryos during the fifth week of maturation; **(EC Vb)** detailed morphology of mature embryos showing a circle of longer cotyledons (C), hypocotyl (H), and radicle (R). **(EC VIa)** Cluster of post-mature somatic embryos during the sixth and seventh weeks of maturation (the arrow highlights a malformed embryo on the surface of the cluster in which the process of callogenesis has begun); **(EC VIb)** detailed morphology of post-mature embryos, with an open crown of long cotyledons (C), elongated hypocotyl (H), and radicle (R); the arrow identifies a malformation in the hypocotyl. **Ia, IIa, IIIa, IVa, Va**, and **VIa**. Scale bar = 5 mm; **Ib, IIb** Scale bar = 500 μm; **IIIb, IVb, Vb, VIb**. Scale bar = 1 mm.

**FIGURE 2:**
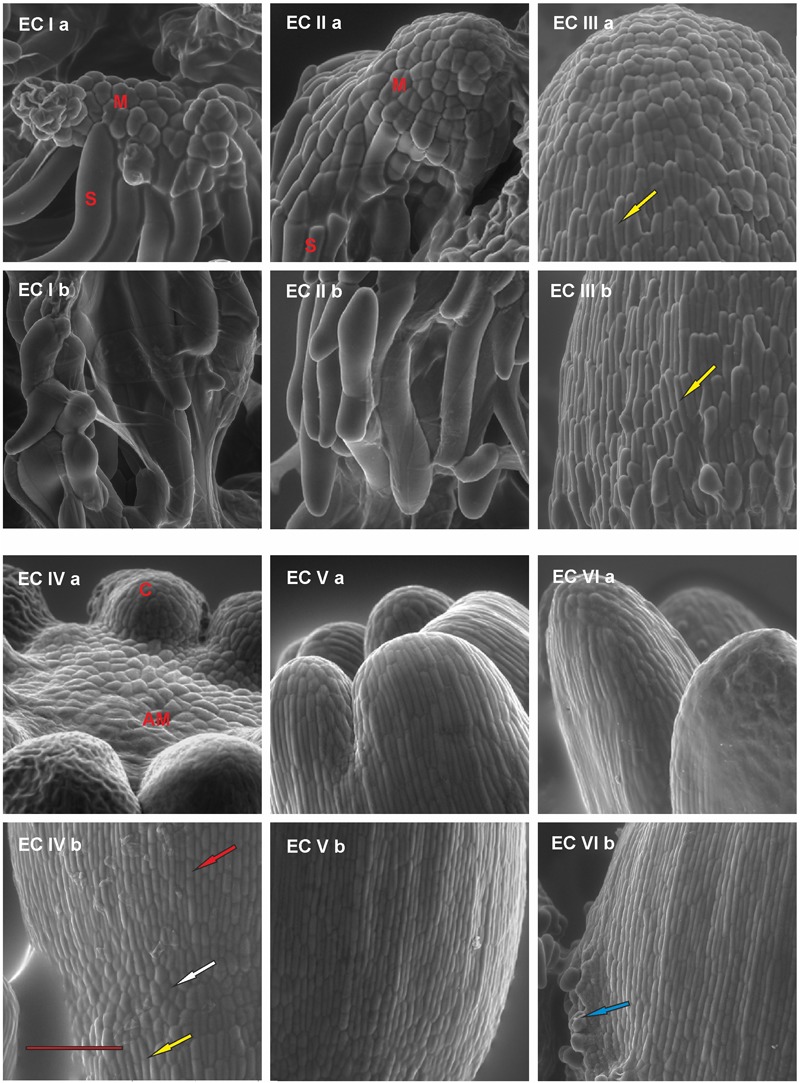
Documentation of the development of the outermost cell layers in *Picea abies* somatic embryos at distinct phases of somatic embryo maturation. **(EC Ia)** Well-arranged meristematic embryonal heads (M) of polyembryogenic center are connected to suspensor cells (S); **(EC Ib)** a suspensor is composed of long suspensor cells. **(EC IIa)** A compact embryonal head (M) covered by polyhedral cells of early protoderm that are typical for globular embryos; **(EC IIb)** elongated suspensor cells, which are distally located from the meristematic embryonal head. **(EC IIIa)** An apical part of the precotyledonary embryo that is covered by polyhedral cells of the protoderm; note the elongated cells of the root cap reaching almost the top of the embryo in the left lower part of the image (yellow arrow); **(EC IIIb)** lower part of the embryo covered by elongated and overlapping cells of the root cap (yellow arrow). **(EC IVa)** Apical meristem of cotyledonary embryo (AM) surrounded by cotyledons (C); **(EC IVb)** junction zone between the hypocotyl and the root cap of cotyledonary embryo; polyhedral cells of protoderm (white arrow) are characteristic of this zone, they are surrounded proximally by elongated cells of hypocotyl (red arrow) and distally by elongated cells of the root cap (yellow arrow). **(EC Va)** Cotyledons of a mature embryo covered by elongated, well-arranged protodermal cells. The polyhedral cells are present on the very tip of cotyledons; **(EC Vb)** well-arranged pattern of elongated protodermal cells on the hypocotyl. **(EC VIa)** Elongated cotyledons of a post-mature embryo; **(EC VIb)** hypocotyl presenting with a sign of callus formation (blue arrow). Scale bar = 200 μm.

The characterization of every stage of embryo development is crucial for determining the extent of stress response as it provides indications of anatomical dissimilarity among the irradiated ECs. The EC contained embryos in different developmental stages over the course of maturation. A detailed assessment of embryo development during each week of maturation enabled us to characterize the EC based on particular developmental stages of predominant embryos as EC I–VI. Six distinct developmental stages, denoted EC I–EC VI, were identified during Norway spruce somatic embryo maturation based on EC morphology, including the constitution of surface layers (Figures 1, 2).

At the start of maturation, the clusters of EC are white and filamentous with macroscopically invisible embryos (Figure 1 – EC Ia). The embryonal heads of early embryos are enlarged and individual embryos begin to release from the polyembryogenic complexes (Figure 1 – EC Ib). The embryonal heads are compact, created from small meristematic cells (Figure 2 – EC Ia) that are linked to elongated suspensor cells (Figure 2 – EC Ib).

During the next step of development, globular and cylindrical embryos predominate in EC: the clusters are similar to that observed during the first stage (Figure 1 – EC IIa), but the polyembryogenic complexes have disintegrated and embryos become visible by naked eye. The embryonal heads continue to elongate (Figure 1 – EC IIb); the bigger embryonal heads of cylindrical embryos have a smooth surface formed by polyhedral cells of the early protoderm (Figure 2 – EC IIa). Suspensors’ complexes are composed of long suspensor cells (Figure 2 – EC IIb).

The third step of development is characterized by rapidly developing precotyledonary embryos that are localized to the surface of EC clusters (Figure 1 – EC IIIa and EC IIIb). The embryonal heads have elongated considerably and the suspensors have disintegrated. This developmental step includes the establishment of new embryonic inner structures as described in our previous studies (Svobodová, 1999; Cvikrová et al., 2016). Apical meristem develops and root meristem, with the root cap, forms at the basal pole of the embryo. The protoderm of the embryo proper is now established and demonstrates a tile pattern (Figure 2 – EC IIIa). The elongated root cap cells cover two-thirds of embryo proper in layers that overlap and resemble the pattern of roof tiles (Figure 2 – EC IIIb).

Cotyledonary embryo development proceeds with the emergence of a ring of cotyledon primordia that grow and differentiate around the apical meristem (Figure 1 – EC IVa and EC IVb; Figure 2 – EC IVa). Tightly connected protodermal cells cover the entire embryo and form its smooth surface. These cells at the tips of cotyledons (Figure 2 – EC IVa) and at the junction zone between the hypocotyl and root cap (Figure 2 – EC IVb) have a polyhedral shape whereas the protodermal cells of the hypocotyl have an elongated shape as they extend parallel to the embryonic axis (Figure 2 – EC IVb).

At the end of maturation, the development of mature somatic embryos morphology is completed (Figure 1 – EC Va and EC Vb). All of the structural parts of the embryo (well-developed cotyledons surrounding apical meristem, hypocotyl, and root meristem covered by the root cap) are now established and no further organ primordia will appear. The elongation of the embryos, as well as cotyledons, continues. Both the cotyledons and hypocotyl of the mature embryos have fully developed protoderm (Figure 2 – EC Va and EC Vb).

Mature embryos are not able to continue their development when cultivation on the maturation media is prolonged, the disintegration process of embryos and callogenesis on embryo surfaces can start (Figure 2 – VIa and VIb).

Ultraviolet-B radiation was applied to ECs in stages I, II, III, IV, and V.

### Viability

**FIGURE 3:**
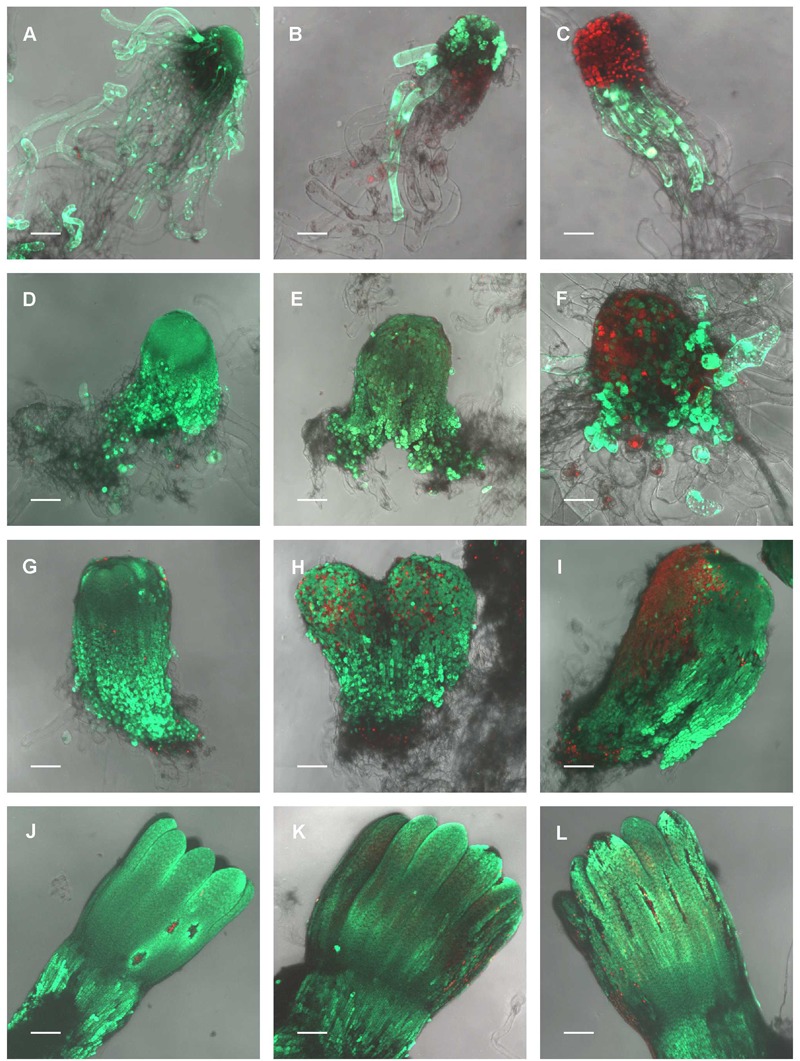
The effect of UV-B radiation on the viability of *Picea abies* somatic embryo cells at distinct phases of somatic embryo maturation. **(A–C)** Control, non-treated embryo, and irradiated embryos at EC I phase of maturation. **(D–F)** Control, non-treated embryo, and irradiated embryos at EC II phase of maturation. **(G–I)** Control, non-treated embryo, and irradiated embryos at EC III phase of maturation. **(J–L)** Control, non-treated embryo, and irradiated embryos at EC IV phase of maturation. First column – control embryos. Second column – embryos on the seventh day after irradiation with 0.1 W m^-2^ h^-1^ UV-B. Third column – embryos on the seventh day after irradiation with 0.6 W m^-2^ h^-1^ UV-B. Green fluorescence indicates living cells (vital staining with fluorescein diacetate), whereas red fluorescence indicates dead cells (staining with propidium iodide). All images are shown at maximum intensity projection of confocal optical sections of the whole embryos; the focal step size between optical sections was 7–16 μm. **(A,D,E,G–L)** Scale bar = 200 μm; **(B,C,F)** scale bar = 100 μm.

In the control embryos, a majority of meristematic cells that forms the embryo proper was viable throughout maturation, although some individual dead cells were observed at all stages of embryo development. On the contrary, the amount of dead suspensor cells increased throughout maturation due to the natural process of programmed cell death (PCD), which is required for normal embryo development (Figures 3A,D,G,J). UV-B irradiation had the largest effect on the viability of somatic embryos in EC I. Exposure to 0.1 W m^-2^ h^-1^ UV-B led to both death of some or all meristematic cells (depending on the size of embryo) and to the release of compact structure of embryonal heads; the suspensor cells were severely injured (Figure 3B). A higher dose of UV-B radiation caused lethal damage to all cells of the embryonal head; suspensor cells were either dead or exhibited signs of disintegration (Figure 3C). Developmentally more advanced cylindrical and precotyledonary embryos (EC II and III) were less affected by UV-B treatment than EC I embryos. Irradiation with 0.1 W m^-2^ h^-1^ UV-B caused the death of only certain individual cells in the embryo surface layers (Figures 3E,H). However, after the application of 0.6 W m^-2^ h^-1^ UV-B, the embryonal heads of EC II embryos were severely injured. Surprisingly, a part of the suspensor cells that were distally located from the embryonal head remained viable even though a majority of the suspensor cells might have already undergone PCD at this stage (Figure 3F). The surface cells of precotyledonary embryos (EC III) that had been directly exposed to radiation exhibited damage, but this was only noticeable at the higher UV-B dose (Figure 3I). Cotyledonary embryos (EC V) were much less affected by UV-B radiation, although individual dead cells could be observed in both hypocotyls and cotyledons (Figure 3K). Exposure to 0.6 W m^-2^ h^-1^ UV-B was more harmful for cells located on the tips of cotyledons and probably caused the ruptures on the hypocotyl surface that were filled with dead cells (Figure 3L).

### Malondialdehyde Content

**FIGURE 4:**
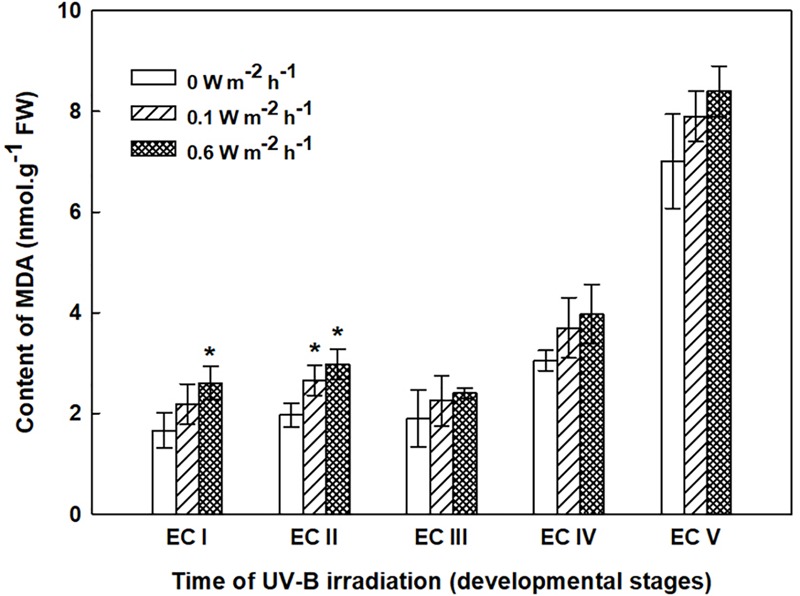
Malondialdehyde (MDA) content in control and irradiated ECs at stages I–V of maturation, determined on the seventh day after UV-B irradiation. Data indicate mean ± SE (*n* ≥ 4). Asterisks above the bars indicate significant differences (*P* < 0.05) between the MDA contents observed in irradiated ECs and the corresponding controls. 0 W m^-2^ h^-1^, control – untreated EC at stages I–V.

Changes in MDA content, determined 7 days after irradiation in EC I–V, are presented in Figure 4. Irradiation of EC I and II with 0.1 W m^-2^ h^-1^ UV-B prompted MDA content to increase by 30 and 35%, respectively, while the MDA contents of the same ECs rose by 60 and 50%, respectively, following 0.6 W m^-2^ h^-1^ UV-B exposure. The MDA contents of EC III and IV increased by 20 and 30% following exposure to 0.1 and 0.6 W m^-2^ h^-1^ UV-B, respectively. All of these changes are reported relative to MDA content in control ECs.

### Polyamine Contents

**FIGURE 5:**
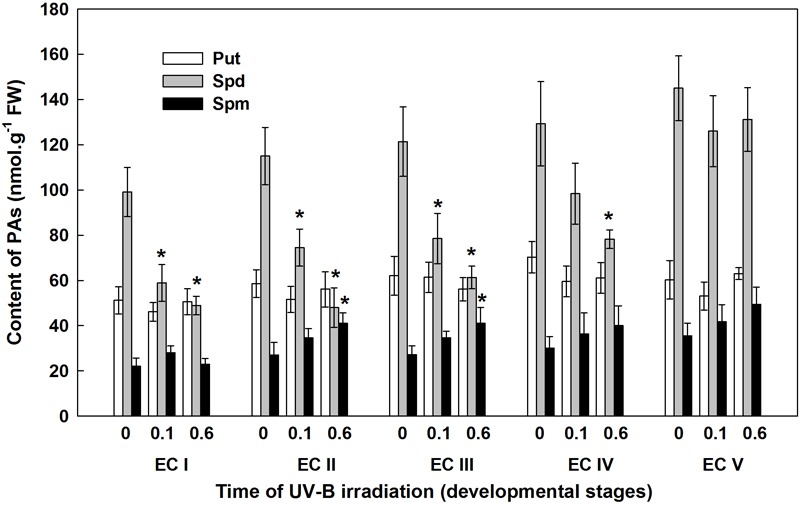
Levels of free polyamines (PAs) in control and irradiated ECs at stages I–V of maturation, analyzed on the seventh day after UV-B irradiation. Data indicate mean ± SE (*n* ≥ 4). Asterisks above the bars indicate significant differences (*P* ? 0.05) between the values observed in irradiated ECs and the corresponding controls. 0 W m^-2^ h^-1^, control – untreated EC at stages I–V; 0.1–0.1 W m^-2^ h^-1^ UV-B; 0.6–0.6 W m^-2^ h^-1^ UV-B; Put, putrescine; Spd, spermidine; Spm, spermine.

Throughout maturation, the spermidine (Spd) content of EC continuously increased; however, statistically significant increase was observed in EC IV and EC V only (results for the controls are indicated with the label “0” in the figures) (Figure 5). Significant differences in Spd content between controls and irradiated EC (both with 0.1 and 0.6 W m^-2^ h^-1^ UV-B) were noted 7 days after irradiation. The Spd content of EC I and II declined approximately 40% following irradiation with 0.1 W m^-2^ h^-1^ UV-B. A higher dose of UV-B, 0.6 W m^-2^ h^-1^, decreased Spd contents in EC I and II by 50 and 40%, respectively. UV-B radiation also had a substantial effect on EC III, with Spd contents in cultures decreasing by about 35 and 50% after exposure to 0.1 and 0.6 W m^-2^ h^-1^ UV-B, respectively. In EC IV, a significant decrease in Spd content was only observed after the culture was exposed to the higher dose of UV-B. All of the reported changes are relative to Spd content in control ECs. In EC II and III, spermine (Spm) levels were substantially higher after the higher dose of UV-B was applied. Only slight differences in the levels of putrescine (Put) were observed in both control and irradiated ECs throughout maturation. The control EC was characterized by a stable Spd/Put ratio, with values ranging from 1.8 to 2.4 (Supplementary Figure S1). The decline in Spd contents observed in irradiated EC I–III markedly decreased the Spd/Put ratio. The Spd/Put ratios of control EC and EC IV that had been irradiated with 0.6 W m^-2^ h^-1^ UV-B differed significantly. EC V demonstrated non-significant changes in the Spd/Put ratio that were irrespective of the applied UV-B dose.

### Phenolic Acid Contents

**FIGURE 6:**
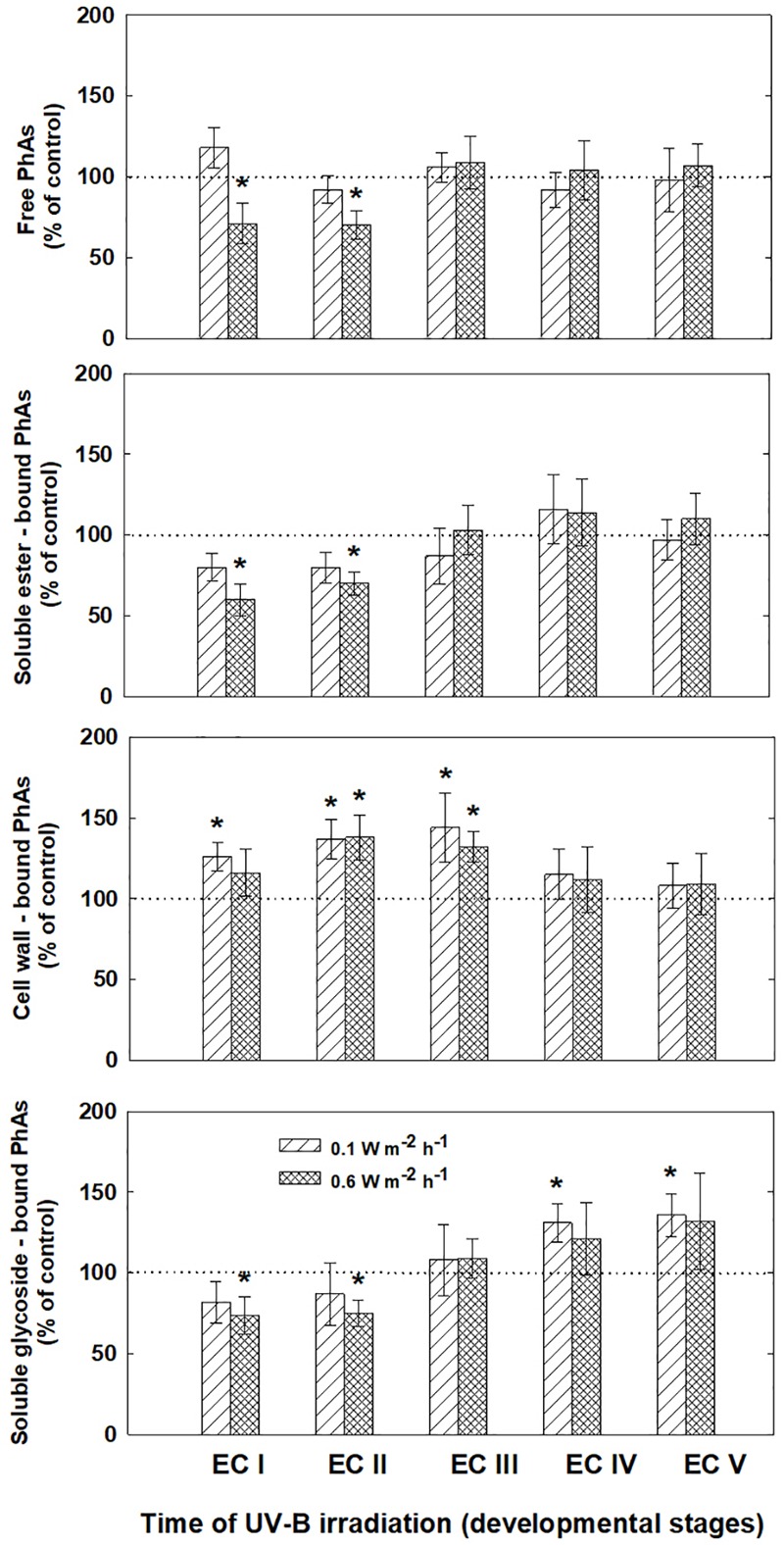
Levels of phenolic acids (PhAs) in ECs irradiated at stages I–V of maturation determined on the seventh day after UV-B application. Changes are expressed relative to the values of untreated EC at stages I–V, which represent 100%. Data are shown as mean ± SE (*n* ≥ 4). Asterisks above bars indicate significant differences (*P* < 0.05) between the contents observed in irradiated ECs and the untreated corresponding controls.

**FIGURE 7:**
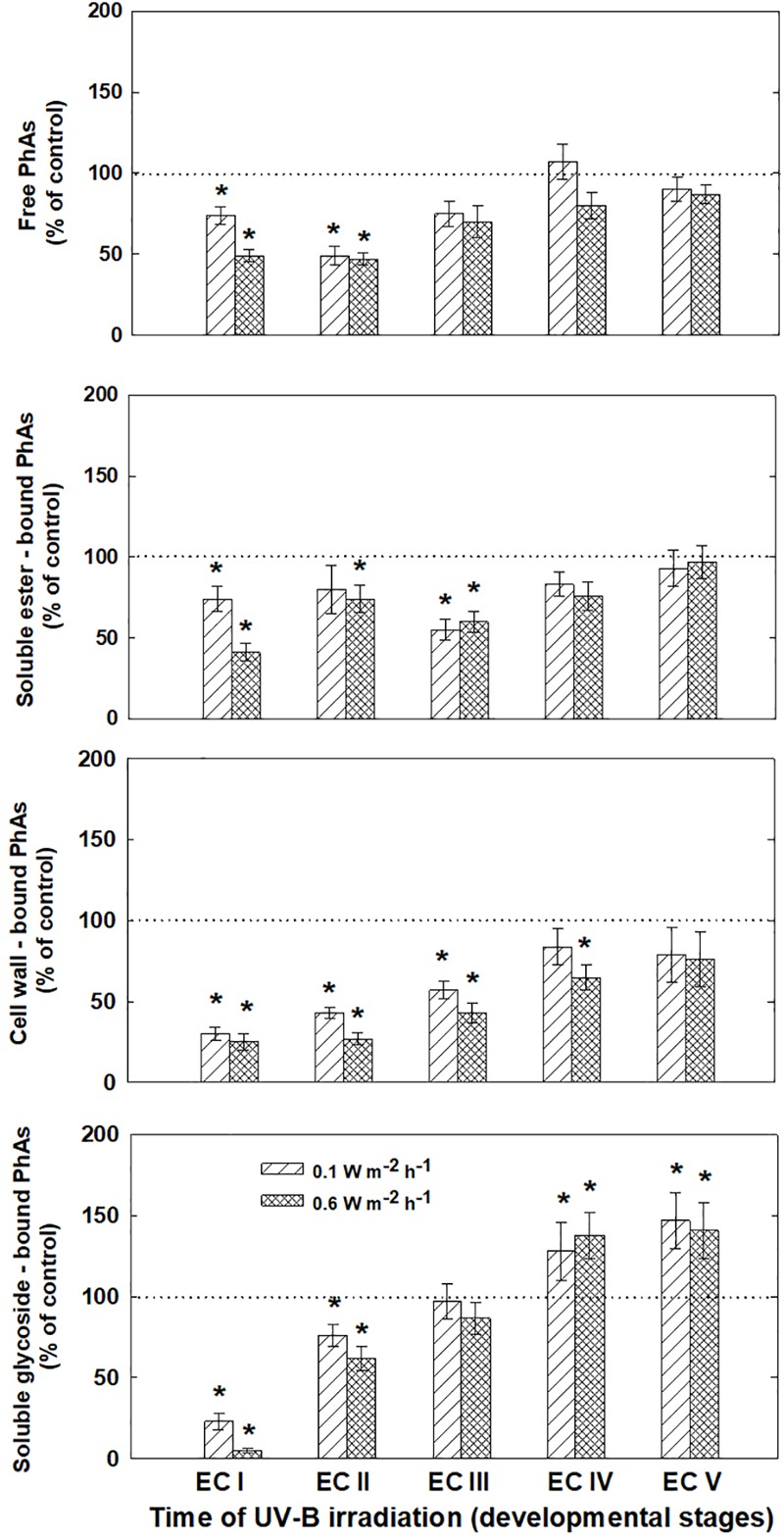
Levels of phenolic acids (PhAs) in ECs irradiated at stages I–V of maturation, determined at the end of maturation. Changes are expressed relative to the values of untreated control EC at the end of maturation, which represent 100%. Data are shown as mean ± SE (*n* ≥ 4). Asterisks above bars indicate significant differences (*P* < 0.05) between the contents observed in irradiated ECs and the untreated control.

**FIGURE 8:**
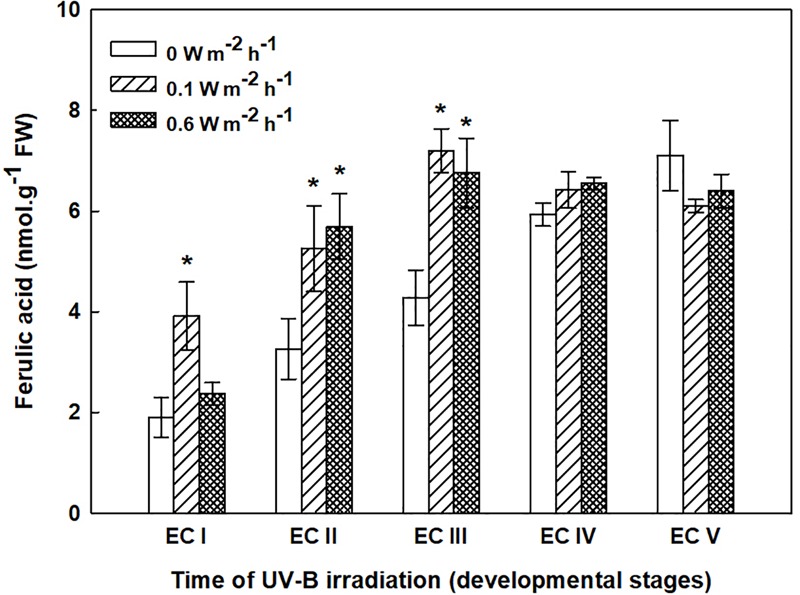
Ferulic acid content in the cell wall-bound fractions from control and irradiated ECs at stages I–V of maturation, determined on the seventh day after irradiation. Data indicate mean ± SE (*n* ≥ 4). Asterisks above bars indicate significant differences (*P* < 0.05) between the contents observed in irradiated ECs and the corresponding controls. 0 W m^-2^ h^-1^, control – untreated EC at stages I–V.

**FIGURE 9:**
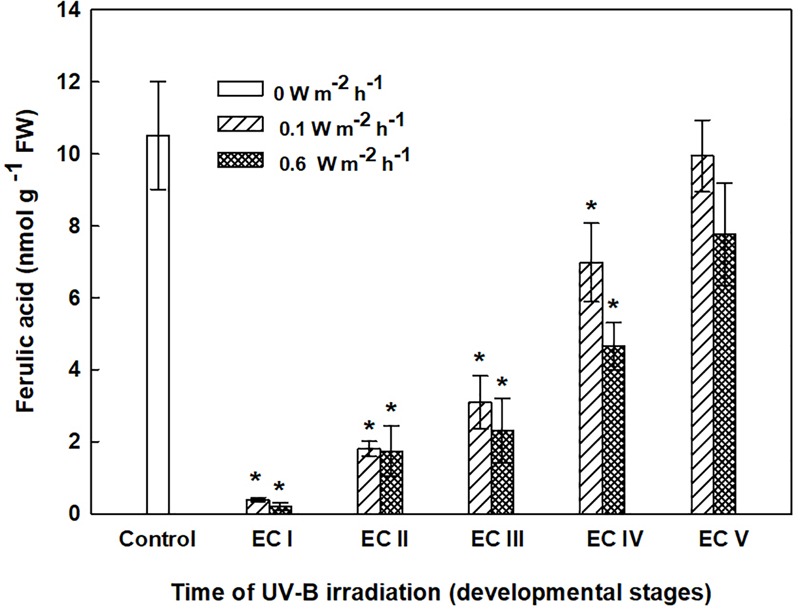
Ferulic acid content in cell wall-bound fractions from control EC and irradiated ECs at stages I–V of maturation, determined at the end of maturation. Data indicate mean ± SE (*n* ≥ 4). Asterisks above bars indicate significant differences (*P* < 0.05) between the contents observed in irradiated ECs and the untreated control. 0 W m^-2^ h^-1^, control – untreated EC at the end of maturation.

**FIGURE 10:**
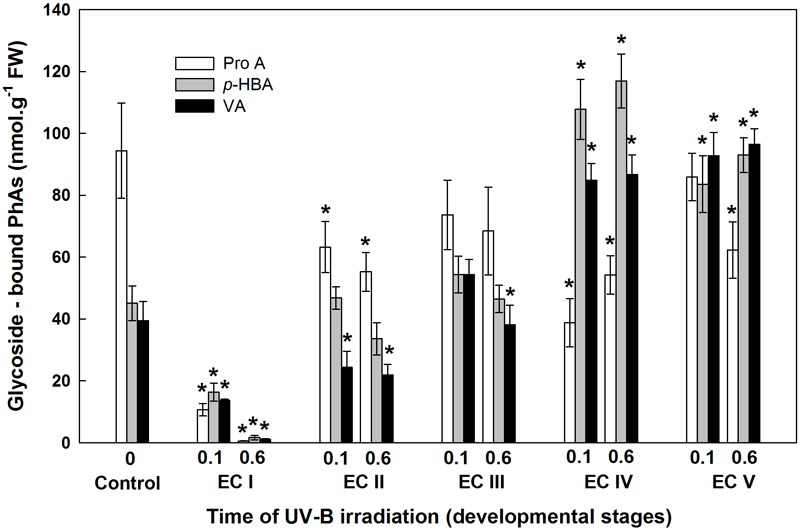
Glycoside-bound methanol-soluble protocatechuic (ProA), *p*-hydroxybenzoic (*p*-HBA), and vanillic (VA) acid contents of control EC and irradiated ECs at stages I–V of maturation, determined at the end of maturation. Data indicate mean ± SE (*n* ≥ 4). Asterisks above bars indicate significant differences (*P* < 0.05) between the contents observed in irradiated ECs and the untreated control. 0 W m^-2^ h^-1^, control – untreated EC at the end of maturation; 0.1–0.1 W m^-2^ h^-1^ UV-B; 0.6–0.6 W m^-2^ h^-1^ UV-B.

The phenolic acids present in ECs I – V were determined on the seventh day after irradiation (Figure 6) and at the end of maturation (i.e., after 6 weeks of maturation, when the yield of embryos was estimated, Figure 7). Analyses revealed that seven phenolic acids were present in the EC extracts: two cinnamic acid derivatives, *p*-coumaric acid and ferulic acid, and five benzoic acid derivatives, *p*-hydroxybenzoic, protocatechuic, vanillic, gallic, and anisic acids. The benzoic acid derivatives extracted by methanol were mainly in free, ester-bound (those released after alkaline hydrolysis) and glycoside-bound (those released after acid hydrolysis, mainly glycosides of *p*-hydroxybenzoic, protocatechuic, and vanillic acids) forms. The most abundant phenolic acid liberated from the cell walls with alkali was ferulic acid, followed by *p*-coumaric acid. Exposure with 0.6 W m^-2^ h^-1^ UV-B clearly decreased free and soluble ester- and glycoside-bound phenolic acids in EC I and II (Figure 6). A significant increase in cell-wall bound phenolic acids was observed in EC I–III 7 days after irradiation (both 0.1 and 0.6 W m^-2^ h^-1^ UV-B treatments) and was mostly a result of elevated ferulic acid levels (Figure 8). No marked differences in phenolic acid contents were observed in EC V following UV-B treatment, with the exception of an increase in the amount of phenolic glycosides, which increased by about 30% relative to the control after exposure to 0.1 and 0.6 W m^-2^ h^-1^ UV-B. The irradiation effect on the levels of phenolics determined at the end of maturation was strikingly different. Significant decreases in phenolic acids, which were dependent on the UV-B dose, were observed in irradiated EC I–III (Figure 7). A noticeable decrease in cell wall-bound phenolics, potentially arising from the pronounced decrease in cell wall-bound ferulic acid, was also observed after EC IV was treated with 0.6 W m^-2^ h^-1^ UV-B. These changes correspond to the failure of somatic embryos to reach advanced stages of development (Figures 7 and 9). In EC IV and V, treatment with both 0.1 and 0.6 W m^-2^ h^-1^ UV-B caused the accumulation of phenolic glycosides, as levels of these compounds increased approximately 30–45% relative to the controls (Figure 7). An increase in the glycosides of *p*-hydroxybenzoic acid and vanillic acid coincided with a decline in glycoside-bound protocatechuic acid (Figure 10).

### Yield of Somatic Embryos

**FIGURE 11:**
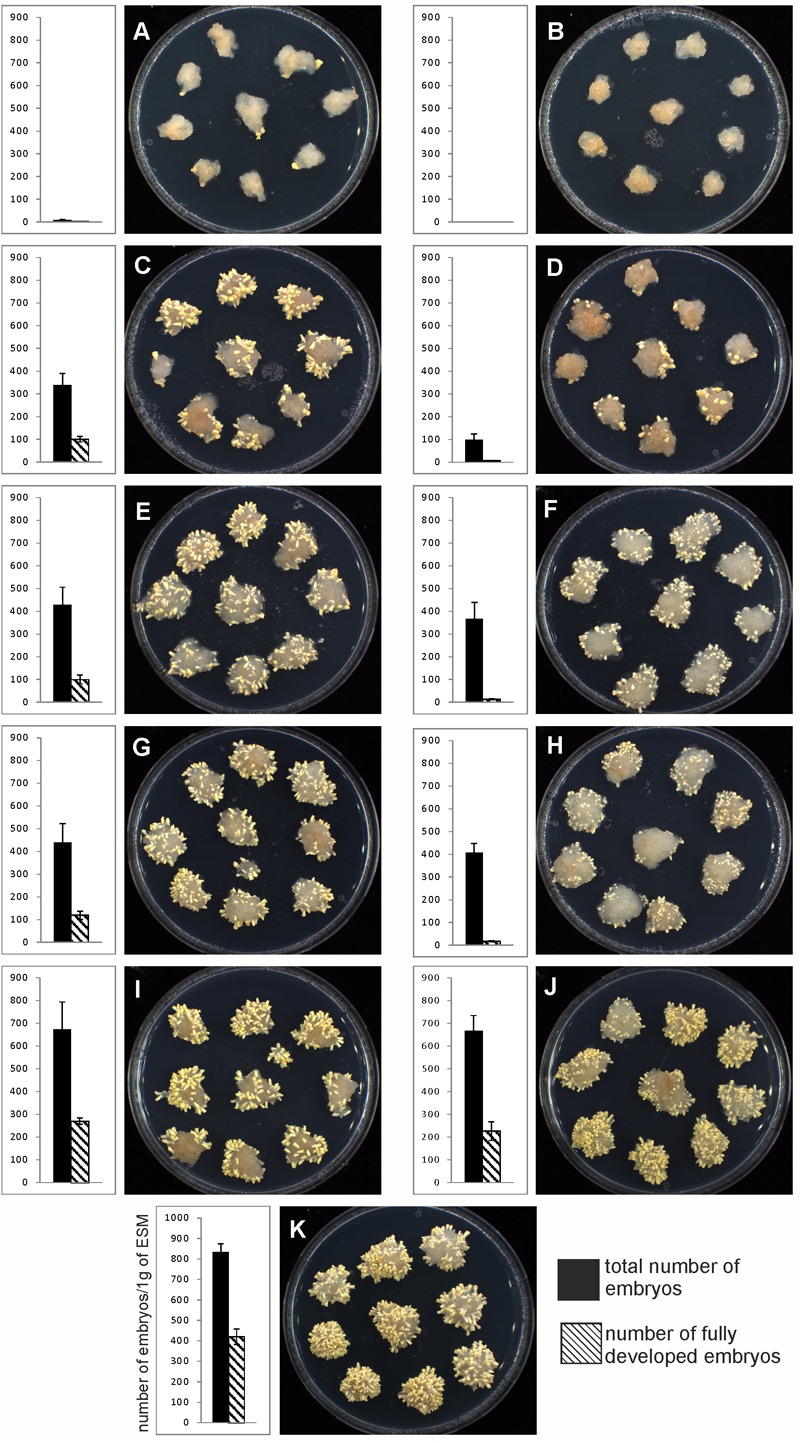
The yield of mature somatic embryos obtained from EC irradiated in distinct phases of maturation. The number of embryos at the end of maturation was expressed on a per-gram basis relative to the fresh mass of embryogenic culture at the start of the experiment. The presented graphs demonstrate the proportion of embryos that are morphologically fully developed (hatched columns) to all embryos (black columns). **(A,B)** UV-B treatment at EC I phase of maturation. **(C,D)** UV-B treatment at EC II phase of maturation. **(E,F)** UV-B treatment at EC III phase of maturation. **(G,H)** UV-B treatment at EC IV phase of maturation. **(I,J)** UV-B treatment at EC V phase of maturation. **(K)** Untreated control. First column – 0.1 W m^-2^ h^-1^ UV-B treatment. Second column – 0.6 W m^-2^ h^-1^ UV-B treatment. The Petri dishes were 90 mm in diameter.

The yield of embryos was estimated after 6 weeks of maturation, i.e., 1–5 weeks after EC irradiation. UV-B treatment affected the total yield of embryos as well as the number of fully developed mature embryos (Figure 11). The most pronounced effect and/or absolute inhibition of somatic embryo development was observed after irradiation of EC I. A marked decrease in the total amount of embryos was also observed in irradiated EC II, as the total amount of embryos reached 41% (lower dose) and 12% (higher dose) of what was observed in the control. Irradiation of EC III and EC IV decreased embryo yield to approximately 50% (lower dose) and 45% (higher dose of UV-B). UV-B treatment only exerted a weak effect on EC V as the total amount of embryos was 80% compared to the control.

Comparing the total number of embryos with the amount of fully developed embryos enabled us to determine how UV-B exposure, as well as its intensity, affects embryo development. In non-treated controls, 50% of all embryos were fully developed. The lower dose of irradiation (0.1 W m^-2^ h^-1^) decreased the number of fully developed embryos to 20–30% in EC II–IV. Treatment with 0.6 W m^-2^ h^-1^ UV-B had an even more pronounced effect; it stopped the further development of irradiated embryos so that only a limited number of fully developed mature embryos could be found [representing only a few (0–7%) percent of all embryos]. Irradiation did not affect EC V severely, as 40% of all the embryos were fully developed embryos, and there was only a small difference between the two UV-B doses (Figure 11).

## Discussion

Our recent results indicate that UV-B light-induced polyphenolic deposition in epidermal cells and specialized idioblastic cells growing within the subepidermal and procambial regions of mature Norway spruce somatic embryos is likely to be part of the protective response to UV-B exposure (Eliášová et al., 2017). The research presented in this paper concentrated on how irradiation affects the development of Norway spruce somatic embryos in terms of the yield of mature embryos and changes in the contents of phenolics and PAs as a defense response to UV-B exposure.

The sequence of morphogenetic events during the development of embryos allowed us to correlate embryo morphology with the stress defense response to UV-B exposure. Generally, a lower dose of UV-B applied to EC I and EC II caused only part of meristematic and suspensor cells to die, whereas a higher dose of UV-B caused either lethal injury to entire embryonal heads or severe damage to individual meristematic cells. However, this result depended on the size of treated embryos. Very small embryos were injured by lower dose of radiation similar to those ones treated by higher dose of UV-B radiation. The reason for the damage of meristematic cells that are mitotically active could consist in their higher sensitivity to UV-B radiation. The fate of suspensor cells is elimination during embryo proper development (Filonova et al., 2002). UV-B radiation might accelerate the process of PCD in these cells. The lesser extent of the damage of some suspensor cells might be related to their less sensitivity and stage of PCD through they underwent. The extent of embryo proper damage could be linked to the differentiation of the outermost cell layers (Eliášová et al., 2017). Protoderm formation begins very early in conifer somatic embryogenesis (Svobodová, 1999; Stasolla et al., 2002) and its establishment is important for further embryo development and maturation (Zhu et al., 2016). Differentiation of Norway spruce protodermal cells proceeded during embryogenesis. Precotyledonary embryos (EC III) were injured on the surface, which was likely the part of the embryo that was directly exposed to UV-B radiation. Cotyledonary embryos (EC IV) and mature embryos (EC V) were affected minimally as the protoderm was already more developed. We can assume that there is some correlation between the process of protoderm formation and the increasing ability of protodermal cells to protect somatic embryos from the harmful effects of UV-B radiation. The outermost cell layer gradually acquire features of mature epidermis; however, this process is only complete after germination (Javelle et al., 2011).

The increase in MDA content, indicating the extent of lipid peroxidation, noted for cultures containing early somatic embryos (EC I, II, and partly III) coincided with changes in the composition of the phenolic substances pool (Figure 4). A decrease in free and ester-bound phenolic acids determined in these cultures (Figure 6) coupled with a significant rise in cell wall-bound phenolics, such as esters of ferulic acid (Figure 8), supports the hypothesis that phenolics incorporated into the cell walls protect cells against UV-B radiation (Schweiger et al., 1996; Semerdjieva et al., 2003). The localization of high levels of phenolic compounds in the epidermal cells of plants subjected to UV-B radiation has been associated with a strategy of protection against UV-B radiation by many authors (Laakso et al., 2000; Martínez-Abaigar et al., 2015). Direct evidence that phenolic accumulation has a role in conferring UV tolerance has been obtained from *Arabidopsis thaliana* (L.) Heynh. mutants containing inactive forms of specific phenolic biosynthetic enzymes (Sheahan, 1996). In conifers, the synthesis of phenolics is crucial to various inducible defense systems. We have recently proposed that the deposition of polyphenolics in intact epidermal cells located in the vicinity of damaged cells may mean that injured cells can transmit a signal to evoke defense response in other tissues (Eliášová et al., 2017). However, the observed increase in glycoside-bound *p*-hydroxybenzoic acid and vanillic acid levels could be considered to be a general antioxidative defense mechanism rather than a specific consequence of UV-B light perception (Figure 10). The observed increases correspond well with results of experiments in which callus cultures of *Pinus sylvestris* were treated with mycelial extracts of *Fusarium nivale* reported by Shein et al. (2003) who, in an earlier contribution (Shein et al., 2001), concluded that the accumulation of *p*-hydroxybenzoic acid plays an important role in the protection of conifer cells.

Significant changes in phenolic substance contents caused by the UV-B treatment of EC I–V, determined at the end of maturation, might be involved in the inhibition of somatic embryo development. Phenolic acids are important to successful embryo development as they are involved in alterations of the cell wall composition during differentiation and morphogenesis (Fry, 1987). Lozovaya et al. (1996) reported that differences between the cell walls of regenerable and non-regenerable callus tissues of *Zea mays, Fagopyrum esculentum*, and *F. tataricum* may be associated with differences in levels and localization of cell wall phenolics. They assumed that alterations in cell wall phenolics during morphogenesis can be used as biochemical markers to demonstrate cell differentiation capacity. A significant decrease in cell wall-bound phenolics arising from the pronounced decrease in cell wall-bound ferulic acid, which was observed after UV-B exposure in EC II–IV, might correspond to failure of somatic embryos to reach more advanced stages of development (Figure 9). We previously found that the cell walls of embryogenic alfalfa calli grown over long periods of time on medium containing glyphosate (an inhibitor of shikimate pathway enzyme 5-enolpyruvyl-shikimate-3-phosphate synthase) show increased amounts of hydroxybenzoic acids, which replaced *p*-coumaric and ferulic acids, and the concomitant gradual loss of calli embryogenic potential (Binarová et al., 1994).

Polyamines have multiple essential functions in plant. They not only facilitate cell division and growth but can also induce cell death by producing H_2_O_2_ as a response to abiotic stress-induced Spd oxidation, which results in the induction of either tolerance responses or the overexpression of certain genes involved in PCD cascades (Moschou et al., 2008, 2012). PAs should no longer be considered only as protective molecules, but rather as multifaceted compounds, which have a key role in the regulation of stress tolerance and are involved in direct interactions with other metabolic routes and hormonal cross-talk (Pál et al., 2015). The accumulation of PAs provoked by UV-treatment has previously been discussed in relation to their role in the prevention of oxidative damage (Lütz et al., 2005; Sfichi-Duke et al., 2008, Schweikert et al., 2011). Several studies have shown that exogenous Spd and Spm applications improve the stress tolerance of plants (Yiu et al., 2009; Hu et al., 2012). As a signaling molecule, H_2_O_2_ subsequently induces expression of antioxidant enzyme genes and their increased activities result in the reduction of oxidative damage. However, the role of PA-derived H_2_O_2_ is still controversial due to its dual functions, and the distinction between the two functions needs to be studied further. Various environmental stresses can cause the accumulation of PAs in plant tissues; however, high stress can lead to a decrease in PA synthesis and may indicate the sensitivity to UV-B radiation (Smith et al., 2001). The significant decrease in the Spd contents of irradiated EC I and II, which contained somatic embryos in early developmental stages, correlated to an increase in MDA levels (Figures 4, 5). On the contrary, a higher dose of UV-B substantially increased the levels of Spm in EC II and III (Figure 5). An increase in MDA and a marked decrease in Spd contents pointed to the fact that early embryos were not well protected against UV-B damage. Alterations in individual PA levels occur in response to a wide variety of stress situations. It has been shown that Put is effective at increasing the activities of antioxidant enzymes (Papadakis and Roubelakis-Angelakis, 2005) while Spd and especially Spm, biologically more dynamic than the other PAs, seem to be involved in preventing injury to membranes under stress conditions (Bouchereau et al., 1999; Kuthanová et al., 2004; Silveira et al., 2004). The relatively high content of Spm (compared with the levels of Put and Spd) as a consequence of cryoprotectant treatment of *P. abies* embryogenic suspensor mass (ESM) also corroborates suggestions that Spm participates in cellular membrane stabilization (Vondráková et al., 2010).

The involvement of PAs in various plant growth and developmental processes, including a crucial role in somatic embryo development, has been reported for several coniferous species (Minocha et al., 2004; Silveira et al., 2004). The variable responses of EC containing early somatic embryos (I and II) and EC containing prevailing cotyledonary embryos (IV and V) to UV-B radiation markedly influenced the yield of somatic embryos (Figure 11). The limited development of embryos caused by UV-B radiation was a result of the decreased viability of irradiated EC I and II and the extent of cell damage in EC III–V, consequences which both coincided with a marked decrease in Spd contents. Spd has been shown to exert a specific morphogenic role in many plant systems. Significant increases in Spd levels were associated with the formation of somatic embryos in *P. abies* (Santanen and Simola, 1992) and *P. radiata* (Minocha et al., 1999) The relationship between embryogenic capacity and the total content of free PAs confirmed that Spd is a crucial part of *P. abies* somatic embryo development and is in agreement with our previous findings (Gemperlová et al., 2009; Vondráková et al., 2015).

## Conclusion

In summary, the exposure of Norway spruce EC to UV-B radiation at different stages of maturation had the strongest effect on cell viability of embryos at early stages of development and resulted in the inhibition of somatic embryo development and/or the substantial decrease in the number of embryos. The extent of cell damage was dependent on the UV-B dose applied, as well as the embryo developmental stage, and might be related to differentiation of the outermost cell layers and formation of protoderm. Developmentally more advanced embryos were superior to early embryos in terms of a more efficient stress defense response to UV-B exposure. The response of early embryos was characterized by an increase in MDA, a marked decrease in PA contents and a decline in phenolics. The reduced ability to activate the defense system seems to be responsible not only for the severe cell damage and decrease in viability but also for the inhibition of embryo development. The significant reduction in Spd, which has been shown to be crucial in the somatic embryo development of several coniferous species, seems to be causally linked to the limited development of embryos. Furthermore, decreased levels of cell wall-bound phenolics arising from the pronounced decrease in cell wall-bound ferulic acid might correspond to failure of somatic embryos to reach more advanced stages of development.

## Author Contributions

ZV, KE, and MC designed the experiments. MV and MC have written the first draft of the manuscript. ZV has participated in collection of plant material for biochemical analysis and has co-written the manuscript. KE has performed and interpreted the data of histological analysis. LG and LF performed the extraction of polyamines and phenolic substances. JM and AT have done HPLC/MS analysis of phenolic acids and polyamines. VN and JR performed the micro-morphological study with ESEM. KE, ZV, and MC wrote the manuscript with contributions from all the authors. MV reviewed the manuscript.

## Conflict of Interest Statement

The authors declare that the research was conducted in the absence of any commercial or financial relationships that could be construed as a potential conflict of interest.
