# Supplementary material for: The Response of Picea abies Somatic Embryos to UV-B Radiation Depends on the Phase of Maturation
Source: Front Plant Sci. 2018 Nov 27;9:1736. doi: 10.3389/fpls.2018.01736 (PMC6277568; doi:10.3389/fpls.2018.01736)
Supplement: Supplementary file 1 [file Data_Sheet_1.PDF]

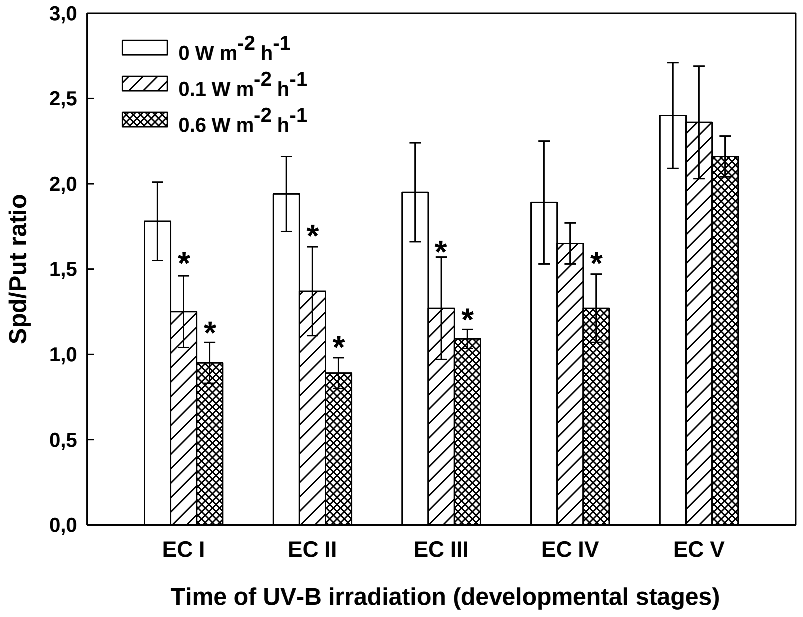

Supplementary Figure S1. Changes in the Spd/Put ratios of control and irradiated ECs at stages I – V of maturation, determined on the 7th day after UV-B irradiation.
Data indicate mean ± SE ($n \geq 4$). Asterisks above bars indicate significant differences ($P < 0.05$) between the values observed in irradiated somatic embryos and the corresponding controls. 0 W m^{-2} h^{-1}, control—untreated EC at stages I – V
